# Supplementary material for: Soluble Receptor for Advanced Glycation End Products (sRAGE) Is a Sensitive Biomarker in Human Pulmonary Arterial Hypertension
Source: Int J Mol Sci. 2021 Aug 10;22(16):8591. doi: 10.3390/ijms22168591 (PMC8395319; doi:10.3390/ijms22168591)
Supplement: Supplementary file 1 [file ijms-22-08591-s001.zip › ijms-1293372-supplementary.pdf]

Supplementary Material

Soluble Receptor for Advanced Glycation End products (sRAGE) is a Sensitive Biomarker in Human Pulmonary Arterial Hypertension

Franziska Diekmann[1], Philippe Chouvarine[1], Hannes Sallmon[2], Louisa Meyer-Kobbe[1], Moritz Kieslich[2], Brian D. Plouffe[3,4], Shashi K. Murthy[3,5], Ralf Lichtinghagen[6], Ekaterina Legchenko[1], Georg Hansmann[1 #]

[1] Department of Pediatric Cardiology and Critical Care, Hannover Medical School, Hannover, Germany
[2] Department of Pediatric Cardiology, Charité University Medical Center, Berlin, Germany
[3] Department of Chemical Engineering, Northeastern University, Boston, MA, USA
[4] Department of STEM, Regis College, Weston, MA, USA
[5] Flaskworks, LLC, Boston, MA, USA
[6] Institute of Clinical Chemistry, Hannover Medical School, Hannover, Germany

KEYWORDS:
Soluble receptor for advanced glycation end products (sRAGE) - pulmonary arterial hypertension – biomarker – vascular injury – inflammation – proliferation – RV hypertrophy

Running Title: sRAGE in human PAH

[#] **Correspondence should be addressed to**:
Prof. Dr. Georg Hansmann, MD, PhD, FESC, FAHA
Department of Pediatric Cardiology and Critical Care
Hannover Medical School
Carl-Neuberg-Str. 1
30625 Hannover, Germany
Email: georg.hansmann@gmail.com
Phone: +49-511-532-9594

TABLE OF CONTENTS

	page
**Abbreviations and Acronyms**	**3**
**Supplementary Text**	4
Supplementary Methods	4
Supplementary Results	5
**Supplementary Figures**	**6**
Figure S1	6
Figure S2	7
Figure S3	8
Figure S4	9
Figure S5	10
Figure S6	11
**Supplementary Tables**	**12**
Table S1	12
Table S2	13
Table S3	14

ABBREVIATIONS and ACRONYMS

6MWD	– six-minute walk distance
AAO	– ascending aorta
AUC	– area under the ROC curve
BMI	– body mass index
BMPR2	– bone morphogenetic protein receptor 2
CI	– cardiac index
CON	– control
CTD-PAH	– connective tissue disease-associated pulmonary arterial hypertension
CTEPH	– chronic thromboembolic pulmonary hypertension
DAMP	– damage-associated molecular pattern
EPPVDN	– European Pediatric Pulmonary Vascular Disease Network
FC	– functional class
GAPDH	– Glyceraldehyde 3-phosphate dehydrogenase
HMGB1	– high-mobility group box 1
HPAH	– heritable pulmonary arterial hypertension
IL-6	– interleukin-6
IMPA1	– inositol monophosphatase 1
IPAH	– idiopathic pulmonary arterial hypertension
LV	– left ventricle
LuTx	– lung transplantation
LVOTO	– left ventricular outflow tract obstruction
mPAP	– mean pulmonary arterial pressure
mSAP	– mean systemic arterial pressure
mTPG	– mean transpulmonary pressure gradient
NTproBNP	– N-terminal pro-brain natriuretic peptide
PA	– pulmonary artery
PAH	– pulmonary arterial hypertension
PASMC	– pulmonary artery smooth muscle cells
PCH	– pulmonary capillary hemangiomatosis
PH	– pulmonary hypertension
PHA	– Pulmonary Hypertension Association
PPARγ	– peroxisome proliferator-activated receptor gamma
PRR	– pattern recognition receptor
PVD	– pulmonary vascular disease
PVOD	– pulmonary veno-occlusive disease
PVR	– pulmonary vascular resistance
ROC	– receiver operating characteristic
RV	– right ventricle
RVH	– right ventricular hypertrophy
RVD	– right ventricular dysfunction
SMC	– smooth muscle cells
sRAGE	– soluble receptor for advanced glycation end products
SVC	– superior vena cava
WHO FC	– World Health Organization functional class
WSPH	– World Symposium on Pulmonary Hypertension

SUPPLEMENTARY TEXT

SUPPLEMENTARY METHODS

Study Population

We focused on female PAH patients in the main manuscript, since registry data in the US and Europe report a female to male sex ratio for adult PAH of 2-4:1 [1-4].

Biomarker Assays

EDTA whole blood samples were immediately centrifuged for 10 min at 1300 g. The plasma was aliquoted and stored at -80°C. N-terminal pro B-type natriuretic peptide (NTproBNP) and Interleukin-6 (IL-6) levels were measured using the Cobas e 801 immunoassay analyzer (Roche Diagnostics, Mannheim, Germany, NTproBNP: REF 07027664190; IL-6: REF 07027532190). Soluble Receptor for Advanced Glycation End Products (sRAGE) levels were determined in adult subjects (no dilution) and pediatric subjects (1:4 dilution) using the Human RAGE Immunoassay (ELISA) (R&D Systems, Minneapolis, MN, USA, DRG00) according to the manufacturer's instructions. Briefly, 100 µl of Assay Diluent was added into the wells of the human RAGE microplate. 50 µl of standard, control or sample was added per well and the plates were incubated for 2 hours at room temperature. The plates were then washed 4 times with 400 µl wash buffer per well. 200 µl of Human RAGE Conjugate was added to each well and the plates were incubated for additional 2 hours at room temperature. After incubation, plates were washed 4 times with 400 µl wash buffer per well. 200 µl Substrate Solution was added per well and the plates were incubated for 30 minutes at room temperature. Finally, 50 µl of Stop Solution was added to each well and the optical density was read at 450 nm with a wavelength correction of 570 nm. Standard curves were determined using nonlinear least squares regression analysis (the nls

function) in R and the sample concentration values were calculated based on the corresponding standard curves.

Human Lung Tissue Sample Collection

Whole human lung tissues were obtained from 17 patients (7 adults and 10 children) who underwent bilateral lung transplantation (LuTx) for end-stage PAH (Table 2, Table S3). Healthy lung tissues (controls) were obtained from 9 LuTx donors (downsizing lungs or unused donor lungs, Table 2). Random peripheral lung tissue not close to the hilus, the main branch pulmonary arteries and main bronchi was obtained from each lung.

SUPPLEMENTARY RESULTS

NTproBNP, IL-6 and sRAGE plasma concentrations are elevated in patients treated with prostacyclins or prostacyclin analogues vs. controls.

Patients treated with prostacyclins or prostacyclin analogues (PCA) for more severe PAH had higher NTproBNP and sRAGE plasma concentrations compared to patients who were not treated with prostacyclin analogues, although this difference did not reach statistical significance. NTproBNP plasma levels of PAH patients who were not treated with prostacyclins (no PCA) were 3.5-fold higher versus controls ($p<0.01$) while plasma concentrations of sRAGE were 1.6-fold higher in the no-PCA samples versus controls ($p<0.01$; **Figure S2A-D**). While there was a significant difference in IL-6 plasma concentrations of patients treated with prostacyclins or prostacyclin analogues versus controls ($p<0.001$), there was no difference in the no-PCA versus control comparison (**Figure S2 C,D**).

SUPPLEMENTARY FIGURES

Figure S1. NTproBNP, IL-6 and sRAGE plasma concentrations in male IPAH patients versus healthy male controls.

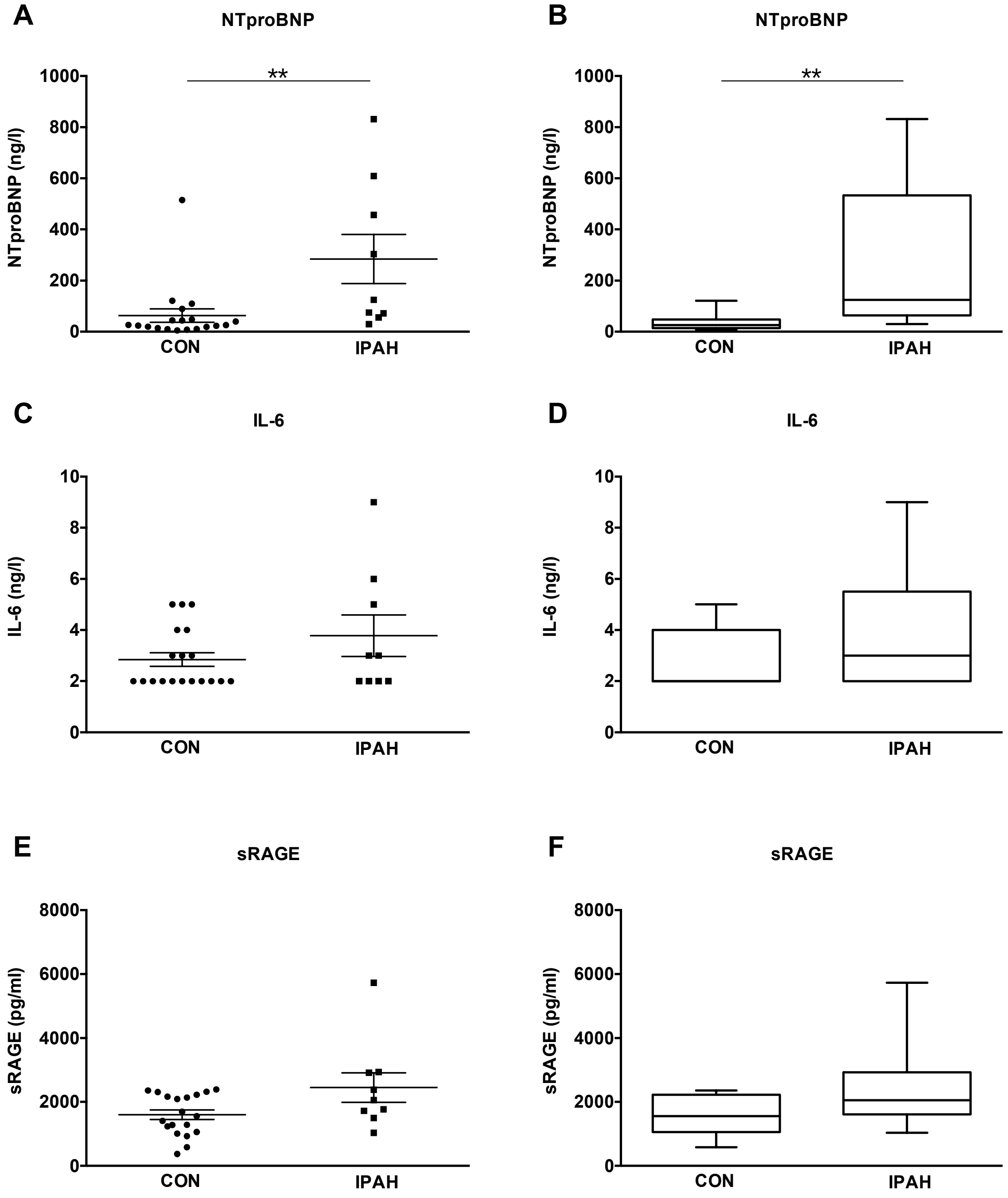

Figure S1. NTproBNP, IL-6 and sRAGE plasma concentrations in male IPAH patients versus healthy male controls. NTproBNP (A and B), IL-6 (C and D) and sRAGE (E and F) plasma concentrations of male patients with IPAH (n=9) vs. controls (n=19). The scatter plots on the left show the mean ± SEM, the box and whiskers plots on the right show the median with interquartile range ± 10-90 percentile. Statistical test: Mann-Whitney U. *p < 0.05, **p < 0.01.

Figure S2. NTproBNP, IL-6 and sRAGE plasma concentrations are elevated in patients treated with prostacyclins or prostacyclin analogues (PCA) vs. controls.

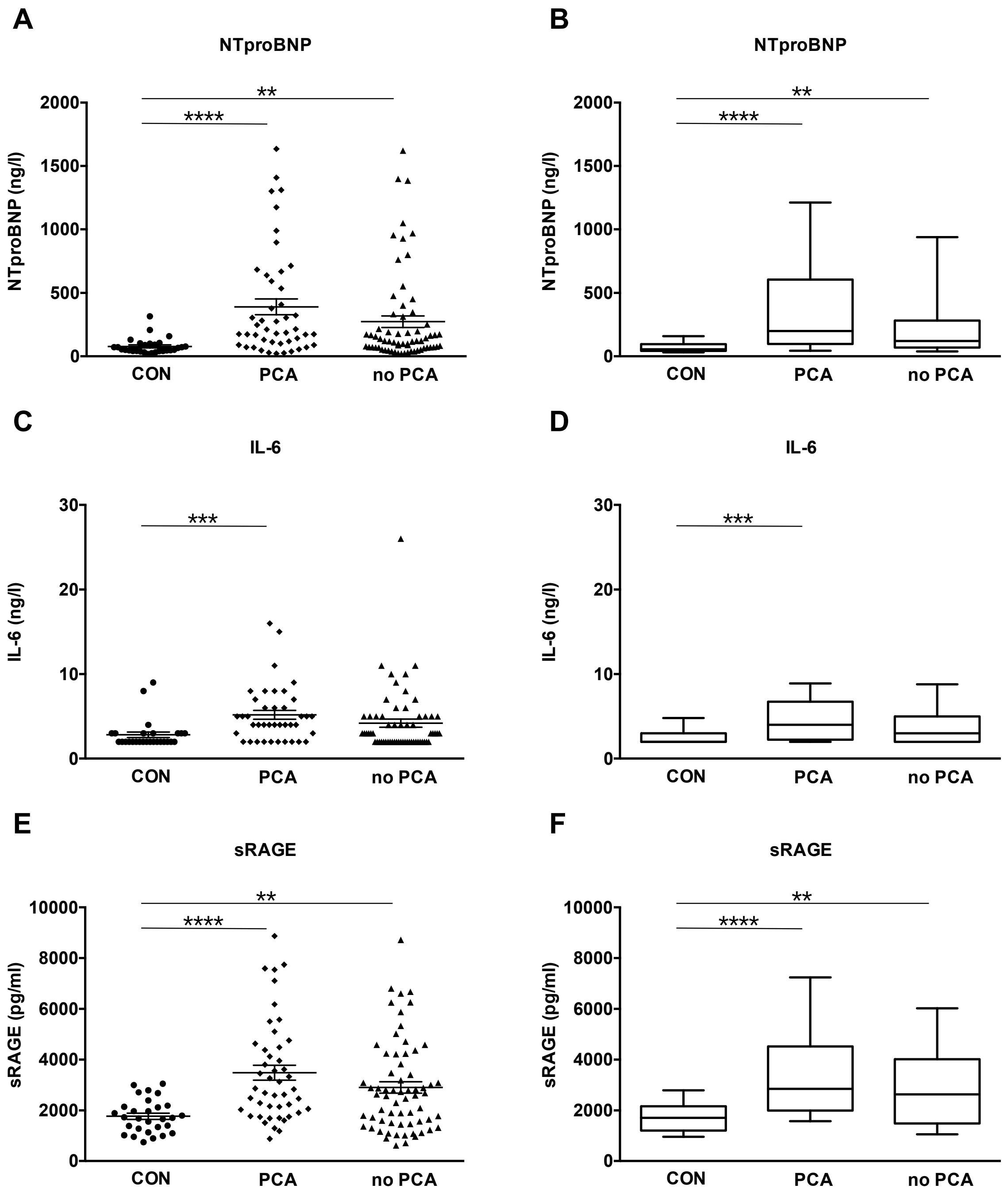

Figure S2. NTproBNP, IL-6 and sRAGE plasma concentrations are elevated in patients treated with prostacyclins or prostacyclin analogues (PCA) vs. controls.
(A and B) NTproBNP plasma concentrations of patients treated with prostacyclins or prostacyclin analogues (PCA, n=46) and patients without prostacyclins/prostacyclin analogues (no PCA, n=65) vs. controls (CON, n=29). (C and D) IL-6 plasma concentrations in PCA (n=40) and no PCA (n=61) vs. controls (n=27). (E and F) sRAGE plasma concentrations in PCA (n=46) and no PCA (n=65) vs. controls (n=29). Statistical test: Kruskal-Wallis test, corrected by multiple testing with Dunn's test. * $p < 0.05$, ** $p < 0.01$, *** $p < 0.001$, **** $p < 0.0001$.

Figure S3. Circulating sRAGE plasma concentrations are not age-dependent in adults.

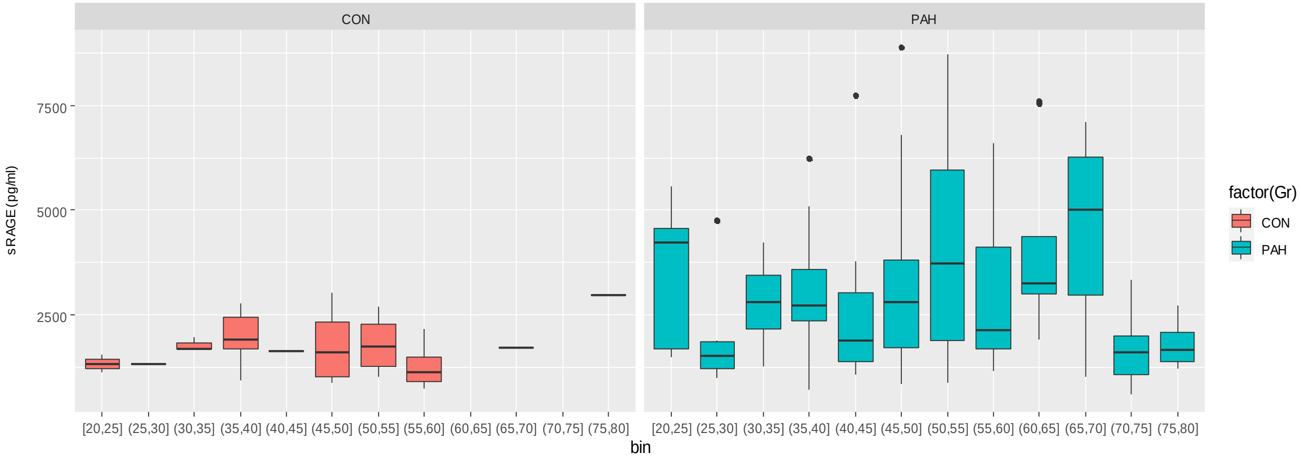

Figure S3. Circulating sRAGE plasma concentrations are not age-dependent in adults. Soluble RAGE is increased in adult PAH patients independently of their age. The box plots show the median, the lower and upper hinges correspond to the first and third quartiles (the 25th and 75th percentiles). The whiskers extend 1.5 * IQR from the hinges. The points beyond the whiskers are outliers. Abbreviations: CON, control; PAH, pulmonary arterial hypertension; sRAGE, soluble receptor for advanced glycation end products.

Figure S4. Correlation between plasma soluble RAGE and NTproBNP in adults.

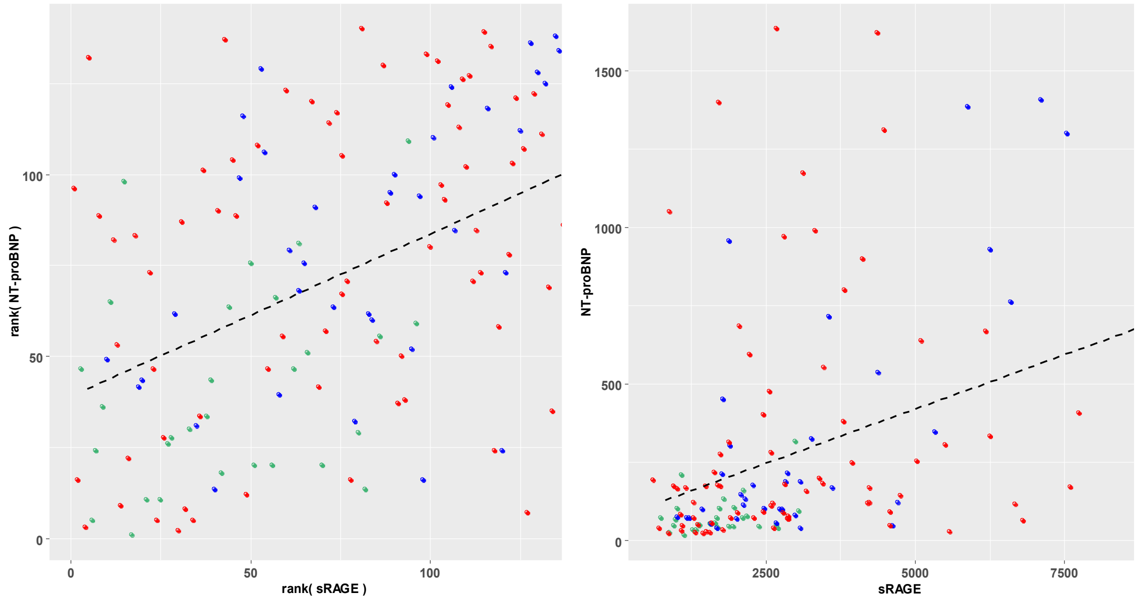

Figure S4. Correlation between plasma soluble RAGE and NTproBNP in adults, measured by enzyme-linked immunosorbent assay. Spearman correlation showing rank NTproBNP vs. rank sRAGE (left) and Pearson correlation NTproBNP vs. sRAGE (right). Since the data did not pass Shapiro-Wilk multivariate normality test, Spearman's rho was calculated (rho = 0.4465, p < 0.0001). The data are color-coded by disease as follows: red = IPAH, blue = CTD-PAH, green = CON). Abbreviations: CTD-PAH, connective tissue disease associated pulmonary arterial hypertension; CON, control; IPAH, idiopathic pulmonary arterial hypertension.

Figure S5. Uncropped full original images of western blots with protein marker.

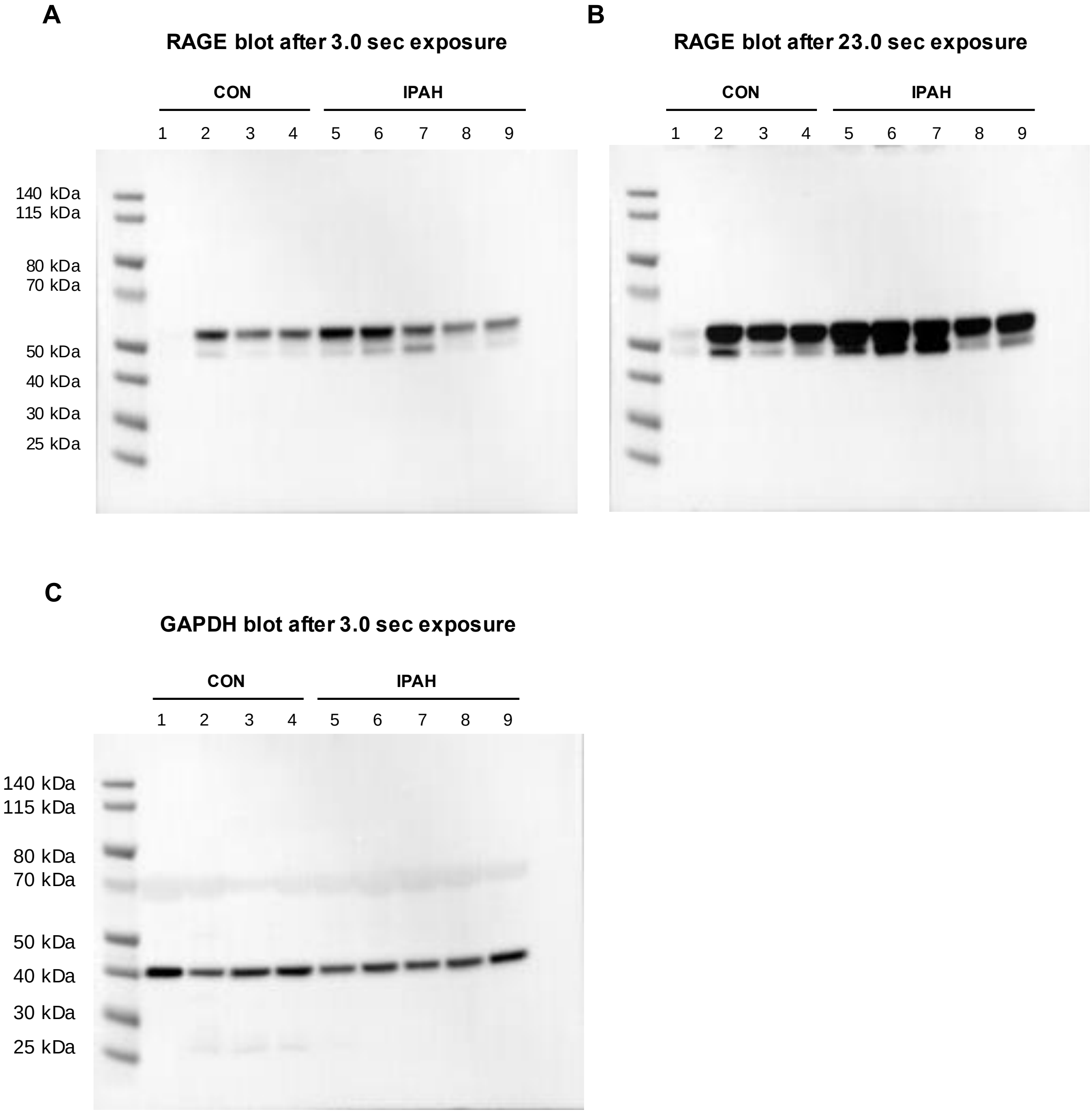

Figure S5. Uncropped full original images of western blots with protein marker. SuperSignal West Pico PLUS Chemiluminescent Substrate Solution was used to visualize the binding of the secondary HRP-antibody. **(A)** The full membrane image of RAGE blot after 3.0 seconds exposure. **(B)** shows that lane one of the control samples becomes apparent after an exposure time of 23.0 seconds, when the other lanes are already saturated. **(C)** The full membrane of the GAPDH blot after an exposure time of 3.0 seconds.

Figure S6. Circulating plasma sRAGE levels in children tend to decrease with age.

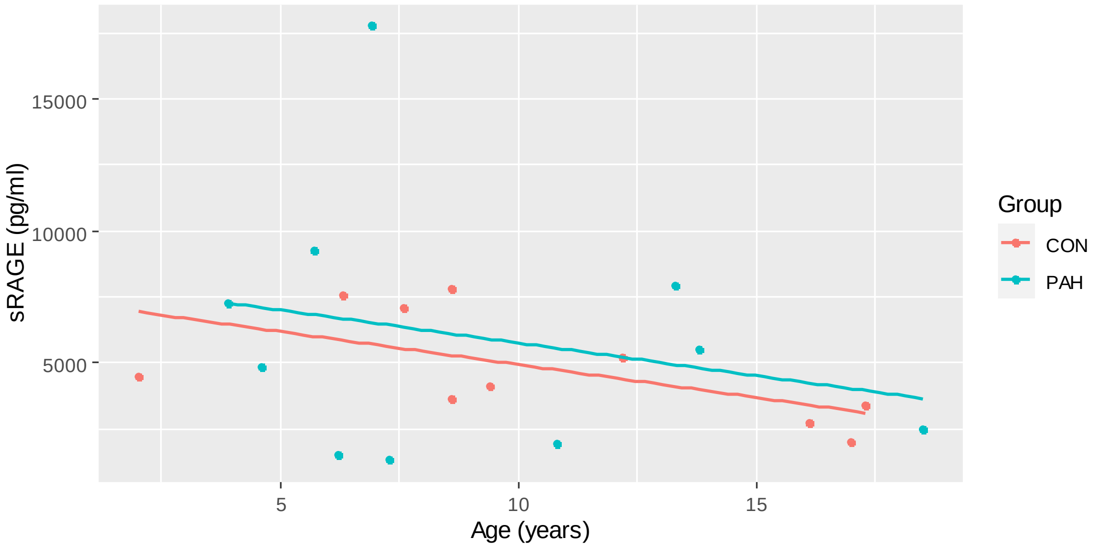

Figure S6. Circulating plasma sRAGE levels in children tend to decrease with age. The regression lines indicate the age-dependent decrease of sRAGE levels (SVC) in both children with moderate PH (n=10; age range 3.9 – 18.5) and children with left ventricular outflow tract obstruction or s/p reconstruction of a double aortic arch (n=10; age range 2.0 – 17.3), measured by enzyme-linked immunosorbent assay in the superior vena cava (SVC).

SUPPLEMENTARY TABLES

Table S1. Characteristics of male PAH patients and healthy controls.

	CON (N = 19)	PAH total (N = 9)	IPAH (N = 9)	CTD-PAH (N = 0)
**Demographics**				
Age – years	50.3 (24 – 80)	49.2 (29 – 72)	49.2 (29 – 72)	
Male sex – n	19	9	9	
Height – m	1.79 ± 0.02	1.75 ± 0.03	1.75 ± 0.03	
Weight – kg	96.6 ± 4.1	82.5 ± 6.0	82.5 ± 6.0	
BMI – kg/m^2	30.2 ± 1.2	26.6 ± 1.5	26.6 ± 1.5	
**Functional Status**				
WHO FC I – n (%)	-	1 (11%)	1 (11%)	
WHO FC II – n (%)	-	6 (67%)	6 (67%)	
WHO FC III – n (%)	-	2 (22%)	2 (22%)	
**Biomarker**				
NTproBNP – ng/l	63.1 ± 26.3	284.3 ± 96.2	284.3 ± 96.2	
**Race/ethnicity**				
White	14	8	8	
Black	3	0	0	
Asian	0	1	1	
Hispanic	2	0	0	
other	0	0	0	

Table S1. *Characteristics of male PAH patients and healthy controls.* Values are presented as number of subjects or as mean ± SEM. Abbreviations: BMI, body mass index; CTD-PAH, connective tissue disease-associated pulmonary arterial hypertension; IPAH, idiopathic pulmonary arterial hypertension; NTproBNP, N-terminal prohormone of brain natriuretic peptide; PAH, pulmonary arterial hypertension; WHO FC, World Health Organization Functional Class.

Table S2. Characteristics of pediatric PH Patients and Non-PH Controls

	PH Patients (N = 10)	Non-PH Controls (N = 10)	p value
**Demographics**			
Age – years	9.1 (3.9 – 18.5)	10.5 (2.0 – 17.3)	n.s. (0.3822)
Male sex – n (%)	4 (40%)	6 (60%)	
Height – m	1.30 ± 0.09	1.39 ± 0.09	n.s. (0.3046)
Weight – kg	30.2 ± 7.2	33.9 ± 4.6	n.s. (0.2713)
BSA – m^2	1.0 ± 0.1	1.1 ± 0.1	n.s. (0.3061)
**Clinical Diagnosis**			
	1.1 IPAH – 3<br>1.2 HPAH – 3<br>1.4.3 Portal hypertension – 1<br>1.4.4 PAH-CHD – 1<br>PH group 3 (lung disease) – 2	LVOTO – 9<br>s/p double aortic arch – 1	
**Functional Status**			
WHO FC	2.5 ± 0.2	1.0	
6 MWD – m, n = 5	344.0 ± 77.6	N/A	
NTproBNP (SVC) – ng/l	168.8 ± 50.8	79.6 ± 12.4	n.s. (0.1594)
IL-6 (SVC) – ng/l	2.5 ± 0.3	2.3 ± 0.2	n.s. (0.8607)
**Risk stratification (EPPVDN)**			
Invasive Risk – n	Intermediate Risk – 9<br>Lower Risk – 1		
Higher Risk score (max 21)	3.6 ± 0.6		
Lower Risk score (max 20)	12.5 ± 0.9		

Table S2. *Characteristics of PH Patients and Non-PH Controls.* Values are presented as mean ± SEM. NTproBNP and IL-6 were measured in superior vena cava (SVC) of all patients. A Mann-Whitney U test was applied. $p < 0.05$ was considered significant. Abbreviations: 6 MWD, six minute walk distance; BSA, body surface area; EPPVDN, European Pediatric Pulmonary Vascular Disease Network; HPAH, hereditary pulmonary hypertension; IL-6, Interleukin-6; IPAH, idiopathic pulmonary hypertension; LVOTO, left ventricular outflow tract obstruction; MWD, minute walk distance; NT-proBNP, N-terminal pro b-type natriuretic peptide; PAH, pulmonary arterial hypertension; PH, pulmonary hypertension; s/p, status post; WHO FC, World Health Organization Functional Class.

Table S3. Human lung tissue samples from pediatric end-stage PAH patients

Group	Gender	Age (years)	Diagnosis
PAH	male	11	PVOD/PAH (group 1.6 PH)
PAH	female	17	IPAH (group 1.1 PH)
PAH	female	16	IPAH (group 1.1 PH)
PAH	female	11	PVOD/PCH/PAH (group 1.6 PH)
PAH	female	6	IPAH (group 1.1 PH)
PAH	female	6	IPAH (group 1.1 PH)
HPAH	female	5	HPAH, BMPR2 mutation (group 1.2 PH)
HPAH	female	8	HPAH, BMPR2 mutation (group 1.2 PH)
HPAH	female	17	HPAH, BMPR2 mutation (group 1.2 PH)
HPAH	female	3	HPAH, BMPR2 mutation (group 1.2 PH)

Table S3. Human lung tissue samples from pediatric end-stage PAH patients. Whole human lung tissues from patients with BMPR2 +/- mutation (HPAH), idiopathic pulmonary arterial hypertension (IPAH) and pulmonary veno-occlusive disease (PVOD) / pulmonary capillary hemangiomatosis. (PCH). Abbreviations: HPAH, heritable pulmonary arterial hypertension; IPAH, idiopathic pulmonary arterial hypertension; PAH, pulmonary arterial hypertension; PCH, pulmonary capillary hemangiomatosis; PVOD, pulmonary veno-occlusive disease.

1. Batton, K. A.; Austin, C. O.; Bruno, K. A.; Burger, C. D.; Shapiro, B. P.; Fairweather, D., Sex differences in pulmonary arterial hypertension: role of infection and autoimmunity in the pathogenesis of disease. *Biol Sex Differ* **2018,** 9, (1), 15.
2. Badesch, D. B.; Raskob, G. E.; Elliott, C. G.; Krichman, A. M.; Farber, H. W.; Frost, A. E.; Barst, R. J.; Benza, R. L.; Liou, T. G.; Turner, M.; Giles, S.; Feldkircher, K.; Miller, D. P.; McGoon, M. D., Pulmonary arterial hypertension: baseline characteristics from the REVEAL Registry. *Chest* **2010,** 137, (2), 376-87.
3. Humbert, M.; Sitbon, O.; Chaouat, A.; Bertocchi, M.; Habib, G.; Gressin, V.; Yaici, A.; Weitzenblum, E.; Cordier, J. F.; Chabot, F.; Dromer, C.; Pison, C.; Reynaud-Gaubert, M.; Haloun, A.; Laurent, M.; Hachulla, E.; Simonneau, G., Pulmonary arterial hypertension in France: results from a national registry. *Am J Respir Crit Care Med* **2006,** 173, (9), 1023-30.
4. Escribano-Subias, P.; Blanco, I.; Lopez-Meseguer, M.; Lopez-Guarch, C. J.; Roman, A.; Morales, P.; Castillo-Palma, M. J.; Segovia, J.; Gomez-Sanchez, M. A.; Barbera, J. A., Survival in pulmonary hypertension in Spain: insights from the Spanish registry. *Eur Resp J* **2012,** 40, (3), 596-603.
